# Super-Exponential Growth in Models of a Binary String World

**DOI:** 10.3390/e25010168

**Published:** 2023-01-13

**Authors:** Marco Villani, Roberto Serra

**Affiliations:** 1 Department of Physics, Informatics and Mathematics, Modena and Reggio Emilia University, 41121 Modena, Italy; 2 European Centre for Living Technology, 30123 Venice, Italy; 3 Institute of Advanced Studies, University of Amsterdam, 1012 WX Amsterdam, The Netherlands

**Keywords:** Theory of the Adjacent Possible, TAP equation, “hockey stick” curve, nonlinear differential equation, simulation model, Gillespie algorithm

## Abstract

The Theory of the Adjacent Possible (TAP) equation has been proposed as an appropriate description of super-exponential growth phenomena, where a phase of slow growth is followed by a rapid increase, leading to a “hockey stick” curve. This equation, initially conceived to describe the growth in time of the number of new types of artifacts, has also been applied to several natural phenomena. A possible drawback is that it may overestimate the number of new artifact types, since it does not take into account the fact that interactions, among existing types, may produce types which have already been previously discovered. We introduce here a Binary String World (BSW) where new string types can be generated by interactions among (at most two) already existing types. We introduce a continuous limit of the TAP equation for the BSW; we solve it analytically and show that it leads to divergence in finite time. We also introduce a criterion to distinguish this type of behavior from the familiar exponential growth, which diverges only as t → ∝. In the BSW, it is possible to directly model the generation of new types, and to check whether the newborns are actually novel types, thus discarding the rediscoveries of already existing types. We show that the type of growth is still TAP-like, rather than exponential, although of course in simulations one never can observes true divergence. We also show that this property is robust with respect to some changes in the model, as long as it deals with types (and not with individuals).

## 1. Introduction

Fast growth processes, which can take place in very different systems, are often described by an exponential function:

(1) $$x\left(t\right)=Ae^{kt}$$

where *A* and *k* are positive constants and *x*(*t*) > 0 is a measure of the “size” of the system at time *t* ≥ 0. Size is used here as an abstract term: it may refer to the actual size of an object or of a region, but also to other measures, for example the intensity of a signal. It often refers to the size of a population of “entities”, like e.g., animals, viruses, molecules or artifacts. In that case, Equation (1) holds if a continuous function can provide an adequate approximate measure of the size of the population (*x* should be always non-negative, *x* = 0 meaning extinction).

As it is well-known, the nonlinear exponential function of Equation (1) is a solution of the linear differential equation:

(2) $$\frac{dx}{dt}=kx$$

with the initial condition *x*(0) = *A* > 0. If *k* > 0 it diverges in the infinite time limit ($\underset{t\to \propto }{\lim}x\left(t\right)=+\infty$).

Note that no true divergence to infinity does actually take place in a finite world; however, the use of diverging equations is commonplace in mathematical physics and, more generally, in mathematical modelling. It is usually justified by observing that the validity of the model, or of some simplifying assumptions, breaks down before the threshold for divergence is approached, so a different model is required in that regime. For example, the model of Equation (2), which leads to unbounded exponential growth, provides only an approximation to a more general case of population growth, like e.g., the one described by the Verhulst equation, which adds to the right-hand side of Equation (2) a rate-limiting, negative term proportional to *x*^2^, thus leading to a logistic growth.

While exponential growth approaches infinity only in the infinite time limit, the possibility of divergence of *x* in finite times has been known since long in dynamical population models [1,2,3,4]. Actually, in 1960 Heinz von Foerster and collaborators published a paper in Science [5] with the somewhat odd title “Doomsday: Friday 13 November 2026”. Of course, doomsday (the day of the end of the world) is a religious rather than a scientific concept, and what the authors actually did was to use a mathematical model to estimate the time when the human population was expected, to become infinite.

The interest for this kind of behavior has been recently stimulated by the proposal of a particular equation, which has been introduced to describe “hockey stick” growth curves, which have been identified in different fields. The prototypical case concerns the behavior in time of the number of different types of artifacts, produced by humans, which grew very slowly for many centuries, then started to increase faster and then, suddenly, the rate of introduction of new types of artifacts increased by several orders of magnitude [6,7]. The same equation has also been suggested to describe, inter alia, the growing complexity of goods, the distribution of descendants in patent data [7], the increasing complexity of atoms and molecules in the universe over 13.8 billion years from no atoms to atoms and to ever larger molecules, and the evolution of the increasing complexity of living species on earth over four billion years [8].

Remarkably, the same equation has been applied in the new field of biocosmology, to estimate the size of the statistical ensemble of possible living microstates on our planet, which can have evolved starting from a set of relatively simple building blocks, i.e., atoms of CHNOPS (carbon-hydrogen-nitrogen-oxygen-phosphorus-sulfur) [9]. Last but not least, it has been speculated that it might be at the basis of a putative fourth law of thermodynamics for non-ergodic systems with an expanding phase space [8].

It has been called the TAP equation by its proponents, where TAP stands for Theory of the Adjacent Possible (it should not be confused with the Thouless-Anderson-Palmer equation in spin glass theory, which is sometimes indicated by the same acronym). The notion of Adjacent Possible is quite general [10,11], and it might be associated to different specific equations. In order to be precise, in this paper we will consider the “canonical” form of the equation as used in [6] (see Equation (3) below) and to its variants described in Section 2. For brevity, we will sometimes refer to the “TAP equation” simply as TAP.

The key idea behind TAP is that new entities are generated by modifying and combining already existing entities. This is particularly clear when different types of artifacts are considered, since it has been proposed that a new type derives from previous types by modifications and recombinations [12]. A given artifact might “generate” (i.e., be the predecessor of) a new type: for example, a clock may generate a watch, or a knife can give rise to a paperknife. However, new artifacts can also have more than one “parent”: for example, a computer and a cell phone can give rise to a smartphone, or a bicycle and an internal combustion engine can be the predecessors of a motorcycle. We stress, once again, that these remarks concern the types, not the single specimens.

According to [6], let x be the number of different types of artifacts, or in general of different types of entities. If time increases in discrete steps, then new entities, which show up at time *t* + 1, can be generated only by combining and modifying those which exist at time *t*, so:

(3) $$x_{t+1}=x_{t}+P\sum_{i=1}^{x_{t}}\alpha_{i}\left(\begin{array}{c} x_{t}\\ i \end{array}\right)$$

with 0 < *α_i_* ≤ 1, for every *i*. *P* > 0 defines the overall time scale. It has been shown that Equation (3) can give rise to super-exponential growth, with a hockey-stick behavior with a sharp jump at finite times (see Section 2 for an example).

Let us immediately point out a major difference with respect to the other, familiar types of equations of population dynamics. When we describe e.g., the growth of the number of new animals of an invading species in an ecosystem, or the number of citizens following a trendy political idea, we are interested in the number of individuals. On the contrary, we know that TAP describes the number of new types of artifacts, not the number of their specimens. In order to qualify as a new type, it is necessary that the newborn be different from all its predecessors. Not every modification of a different type, nor every combination of types, counts as a new entity, unless it is different from all the previous ones.

Of course, Equation (3) provides an oversimplified model of the actual processes which take place in nature or in society, but it might capture some essential features, and it has been claimed that TAP can accurately describe the hockey-stick type of behavior in time of the number of different types of artifacts available to humans. We refer the reader to the papers [6,7] for further details. The limitations due to finiteness will sooner or later enter the scene, but they might show up only after a fairly long phase, which is well described by TAP.

Note that types never die in Equation (3). It might be argued that this is unrealistic, so one might consider adding linear death terms like -*μx_t_* to its right-hand side (as for example in [4]). If the death term is so high that it does not allow any initial growth, then of course the behavior would be different, but not particularly interesting. If this is not the case, such a death term can slow down the initial growth, but it does not prevent super-exponential growth, which is due to the higher order terms in the expansion of the right-hand side.

A critical remark on Equation (3) is that its coefficients do not take into account the age of the artifacts. Consider a type which is present at the very beginning of the process; in every time step, it has the same probability of generating something new, and this may be unrealistic, leading to overestimation of the speed of growth of the number of different types [13].

The generation of new artifact types is a complex process, which involves sophisticated cognitive processes as well as social skills [14]. Moreover, quantitative comparison with actual data is complicated by several issues (what qualifies as a new type?). Therefore, in order to understand the main properties of TAP, it is useful to focus on a simpler case, which can be more easily handled. Moreover, it will be particularly interesting to consider a case where recently born types are more likely to generate new types, with respect to older ones, and to check whether super-exponential growth can take place also in these conditions.

In order to define an interesting case, it is necessary to describe both the entities which exist (or can come into existence) and their generation processes. Here we will consider a Binary String World (BSW), composed of binary strings, which can be generated either by the breakup of a longer one, or by the joining of shorter strings. This picture is loosely inspired by the “binary polymer” model related to the origin of life [15,16,17], but it departs from it in several respects. In the original binary polymer model, only catalyzed reactions are allowed, while in our case there is no notion of catalysis. While BSW is not meant to be a model of chemical reactions, the kinship relationship with chemistry will sometimes show up in the choice of the words: so strings will sometimes be referred to as “polymers” and transformations as “reactions”. The “polymers” of the model are linear strings of symbols belonging to a binary alphabet, e.g., {A,B}. Starting from an initial population, new polymers can be generated by two types of “reactions”, i.e., operators like cleavage (which breaks a linear chain into two parts) and condensation (which forms a new polymer by chaining two existing ones).

The Binary String World has the interesting property that new entities can be obtained only by modifications or combinations of existing ones, so it nicely fits the conditions requested by the TAP approach. However, since in the BSW only descendants of a single parent (through cleavage) or of two parents (through condensation) are allowed, while higher order combinations are absent, it is necessary to restrict the sum on the right-hand side of Equation (3) to terms up to second order in x. Moreover, some specific modifications must also be introduced in the way of counting new possible types. Condensation depends upon the order in which strings are chosen, and it is also possible to chain a (type of) string with the same type (see Section 2 for details). The restriction of the canonical TAP to the BSW case, which will be called BTAP, will be given in Section 2, where its continuous-time version will be introduced, and its analytical solution will be provided. It will also be shown that, in spite of the limitation to no more than two parents, it may actually lead to super-exponential growth, diverging at finite times.

However, in the case of the BSW we can also consider what happens if we directly simulate the dynamics of a model of the process: we will make use of an asynchronous stochastic model, where at each time step only a single reaction takes place, chosen at random among those which are possible at that time (i.e., those whose reactants are present in the system at time *t*). This model will be called BSSM (Binary String Species Model), it will be described in detail in Section 3 and the simulation results will be shown in Section 4.

Simulations are necessarily finite; therefore, even when there is a steep growth curve, no true divergence can be observed in BSSM. However, we provide in Section 2 a simple criterion which allows us to distinguish a TAP-like growth curve, with a steep jump at finite times, from an exponential one, which diverges in the asymptotic time limit. In this way we can compare, on a finite time length, the behavior of the simulation model with those of TAP and exponential equations. Note that in the BSSM model it is possible to check whether a newborn polymer type is indeed a new type, thus excluding from the count of novelties all those types which had already appeared earlier—while this is not done in TAP, as pointed out above. By applying the criterion of Section 2 to the simulation results we can compare it to TAP and exponential and verify that it resembles the former (see Section 4). We think that this result, concerning the robustness of super-exponential behavior when the novelty of newborn types is explicitly verified, as the main result of this work, and we will make further comment on it in the final Section 6.

The availability of a dynamical model allows us also to explore how sensible the shape of the growth curve can be with respect to some changes in the model itself. In order to explore this property, we modified it in two ways, which make it closer to a chemical reaction model. An interesting case, which will be called here BSSM2, is similar to the BSSM described above and in Section 3, but differs by the choice of the reaction to be performed, which is no longer done with uniform probability, but in a way which depends upon how many times the reactants have been produced by different reactions (thus resembling in some way the “law of mass action” of chemical kinetics). We will assume that to each string type a number is associated, which is increased whenever a reaction generates a product of the same type—so the numbers provide estimates of the “quantities” of the corresponding strings. If we assume that the reactions take place in a fixed-volume container, then quantities are proportional to densities and the two can be used interchangeably. The choice of the next reaction to be performed then depends also upon these quantities: the probability of choosing a particular cleavage is proportional to the quantity of the single parent polymer, while the probability of choosing a particular condensation is proportional to the product of the quantities of the two parents.

In BSSM2, reactant types do not disappear, and, therefore, it is not a model of chemical kinetics. While no true divergence can of course be observed in finite simulations, it is very interesting to note, as is done in Section 5, that the number of different types grows in a way which indeed resembles that of TAP, according to the criterion which had been introduced in Section 2. This behavior is, therefore, robust with respect to this model change.

A further change has also been considered, which makes the model closer to chemical kinetics, assuming that reactants disappear, i.e., that the quantities of those types which are involved in a reaction actually diminish. In this way, the model, called BSQM (Binary String Quantitative Model), deals no longer with types, but rather with individual molecules (or strings), and the familiar pattern of growth up to a finite saturation level is observed (with oscillations due to the stochasticity of the model), as shown in Section 5.

In the final section, Section 6, we will comment on the results and indicate prospects for future research.

## 2. The Restriction of the TAP Equation to the Two-Parents Case (BTAP)

We are interested in the behavior of the process described in Section 1, where every new type has at most two predecessors; therefore, we will limit the study of Equations (1)–(3) to the first two terms on the right-hand side. Note that, as already anticipated, two modifications are necessary to deal with the BSW:

- The combinatorial terms of the “canonical” Equations (1)–(3) regard the product of the combination of artifact *i* with artifact *k* as identical to the combination of artifact *k* with artifact *i*. The order is immaterial; therefore, the number of different pairs is proportional to $\left(\begin{array}{c} M\\ 2 \end{array}\right)=\frac{M\left(M-1\right)}{2}$ However, this is not the case for the condensations in the Binary String World, where the order of the two terms matters (e.g., the combination of AAB and BAB, i.e., AABBAB, differs from that of BAB and AAB, i.e., BABAAB) so no division by 2 is required. Moreover, it is possible to generate a new string type by condensing a type with itself, so the number of pairs which must be considered is *M*^2^, not *M*(*M* − 1).
- In each cleavage two new strings are created—so, a factor of 2 must be introduced.

Therefore, the BTAP equation is

(4) $$x_{t+1}=x_{t}+P\left(2\alpha_{1}x_{t}+\alpha_{2}{x_{t}}^{2}\right)$$

In Equation (1) *x* is a non-negative integer. If it can be approximated by a continuous variable, and if the time scales of the processes allow one to regard also time as continuous, and if we further suppose that *x*(*t*) is a differentiable function, then one may also consider the continuous-time limit of Equation (4), i.e.,

(5) $$\frac{dx}{dt}=P\left(2\alpha_{1}x+\alpha_{2}x^{2}\right)$$

which can be analytically integrated; its variables can be separated, so:

(5a) $$\int_{x_{0}}^{x}\frac{dx}{2\alpha_{1}x+\alpha_{2}x^{2}}=Pt$$

therefore,

(5b) $$ln\frac{x}{2\alpha_{1}+\alpha_{2}x}-ln\frac{x_{0}}{2\alpha_{1}+\alpha_{2}x_{0}}=2\alpha_{1}Pt$$

Let us define:

(6) $$K_{0}\equiv \frac{x_{0}}{2\alpha_{1}+\alpha_{2}x_{0}}$$

In order for the growth to start, it is necessary that *x*_0_ > 0, so also *K*_0_ > 0. One finally finds:

(7) $$x=\frac{2\alpha_{1}K_{0}}{e^{-2\alpha_{1}Pt}-\alpha_{2}K_{0}}$$

**Figure 1:**
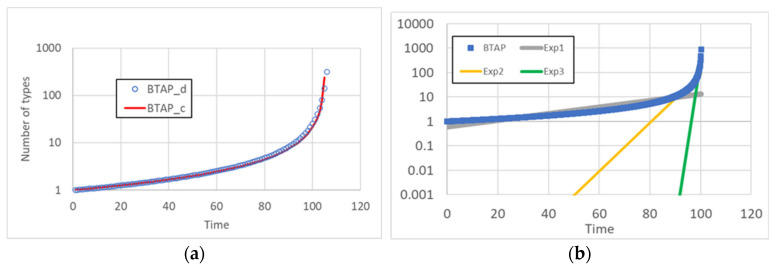
(**a**) Time behavior of the continuous Equation (7) (red curve) and of the discrete version Equation (4) (blue circles). The parameter value is *α*_1_ = 0.1, *α*_2_ = 0.9, P = 0.01 (discrete simulation), and P = 0.0095 (continuous case). The slight difference between the values of continuous P and discrete P is due to the difference between discrete computation (in which dynamics occur in finite steps of equal size, independently of the number of reactions involved) and continuous dynamics. (**b**) The exponential function is unable to follow the trend of the BTAP in its whole extension; in fact, if the slow initial part is well approximated, the fast final ascent is not well approached, while a good approximation of the final ascent does not allow a proper approximation of the initial part. We recall that the approximation of the final part is in any case only partial because the exponential function does not diverge in finite time.

One can directly verify that *x*(0) = *x*_0_ > 0. x starts positive, then it grows, since the denominator becomes smaller and smaller, due to the decrease of the positive term. The denominator approaches 0 as *t* → t*, given by:

(8) $$t=t^{*}=-\frac{1}{2\alpha_{1}P}lnK_{0}=\frac{1}{2\alpha_{1}P}ln\frac{2\alpha_{1}+\alpha_{2}x_{0}}{\alpha_{2}x_{0}}$$

a point in time where *x* diverges. The time behavior of Equation (7) is shown in Figure 1, together with the results of the simulation of the discrete BTAP of Equation (4). It can be observed that the two curves are very similar for large enough values of *x*, at times which are much larger than the discrete time step.

In order to understand the behavior of the simulation of the BSSM, it may be useful to distinguish this TAP-type of divergence at finite times from the usual exponential divergence. It is then helpful to consider the function:

(9) $$y\equiv \frac{x}{2\alpha_{1}+\alpha_{2}x}$$

from Equation (5b) one directly derives:

(10) $$y\equiv \frac{x}{2\alpha_{1}+\alpha_{2}x}=\frac{x_{0}}{2\alpha_{1}+\alpha_{2}x_{0}}e^{2\alpha_{1}Pt}=K_{0}e^{2\alpha_{1}Pt}$$

so the time behavior of *y*(*t*) is exponential, if the growth of *x* is TAP-like.

If *x* increases instead in an exponential way, i.e.,$\;x\left(t\right)=x_{0}e^{kt}$, then:

(11) $$y\equiv \frac{x}{2\alpha_{1}+\alpha_{2}x}=\frac{x_{0}e^{kt}}{2\alpha_{1}+\alpha_{2}x_{0\;}e^{kt}}=\frac{x_{0}}{\alpha_{2}+2\alpha_{1}e^{-kt}}$$

the growth of *y* is logistic, and *y* → *x*_0_*/α*_2_ as *t* increases.

Therefore, the two behaviors can be distinguished by analyzing the time behavior of *y*, which is exponential (whose important characteristic for our purposes is the absence of upper bound) if *x* is TAP, and logistic (which has upper bound) if *x* IS exponential. This analysis must be carried on with care, since it can be complicated by the fact that, before saturation effects show up clearly, a logistic can look similar to an exponential for quite a long time.

## 3. BSSM: The Binary String Species Model

In the Binary String World, the entities are the different types, or “species”, of binary strings (often also called “polymers”, for reasons discussed in Section 1). Each species is an oriented linear chain formed by symbols from the binary alphabet {A,B}, where AB ≠ BA. According to the rules of the BSW, there are two different types of “reactions”, i.e., transformations that can give rise to new strings:

1. condensation, i.e., two reactant species are concatenated forming a longer one (e.g., A + BB → ABB); and
2. cleavage, i.e., a single reactant is split into two shorter products (e.g., ABB → A + BB, or → AB + B);

Obviously, a species can be created by different reactions (e.g., B + BA → BBA and ABBA→A + BBA). A similar system has been studied also in [15,16,18,19], where, however, only reactions catalyzed by another polymer were allowed. No catalyst is involved in the BSSM.

In this and the following section, the “quantity” of each species (string type) is a binary variable, where “0” stands for “absent species” and “1” for “present species”. Once a string species has been created, it remains present in the system, no matter whether it is used as a “reactant” in some reaction. This is coherent with the interpretation of the model entities as types, which can come into existence during the unfolding of the dynamics. In Section 5 we will consider two different cases where the quantities are no longer binary.

The dynamics is defined as follows: each species can undergo cleavage, with a fixed probability per unit time *p_cl_,* and each ordered pair of species can undergo condensation, with a fixed probability *p_cn_* per unit time. In principle, these probabilities can take different values for different species, but in this work, we assume that all the cleavages have the same probability per unit time to occur, and that all the condensations occur with the same probability per unit time (which may be different from that of cleavages). If time is discrete, this gives rise to an equation with the same form as Equation (4). As we have seen, the BTAP equation is based on the assumption that every reaction can produce new entities, while here we can simulate the model and check whether the offsprings are actually different from pre-existing types; of course, only truly new types that have never appeared before, will contribute to the count of *x*.

The time-discrete original TAP is synchronous (all the values are updated at the same time), and this implies that the time step is large enough to accommodate several different reactions. But the same species might be generated by two different reactions, and the synchronous update will be unable to identify it as already existing. While some ad hoc corrections can be envisaged, there is an elegant solution, well-known in chemical kinetics as the Gillespie algorithm [20,21], which is based on choosing a time interval so small that a single reaction occurs in that interval. The duration of the Gillespie time interval Δ*t*, thus depends upon the number of reactions which, in the corresponding synchronous dynamics, would take place simultaneously. In general, the more species that are present, the smaller Δ*t*.

The model is simulated as follows: at each time *t*, a single reaction takes place, chosen at random among all the cleavages and condensations, which are possible in the population *P*(*t*) of strings at that time. If this reaction generates a new type, its result is added to *P*(*t*), generating *P*(*t* + Δ*t*). The binary numbers never decrease: if a string type is already present, its value remains 1, while if a species was absent, and is created at time *t*, its number changes from 0 to 1. Then the new value of Δ*t* is calculated, and the algorithm is iterated. A precise description of the BSSM is given in the Appendix A.

## 4. BSSM Simulation Results

We simulated the model described in Section 3, starting from a state where all the binary polymers of lengths 1, 2 and 3 are present, so the initial condition comprises 14 different types. Our figures often have time on the *x*-axis: this is the “physical” time, analogous in principle to that of the synchronous TAP equation, and not the (usually much smaller) Gillespie step size.

**Figure 2:**
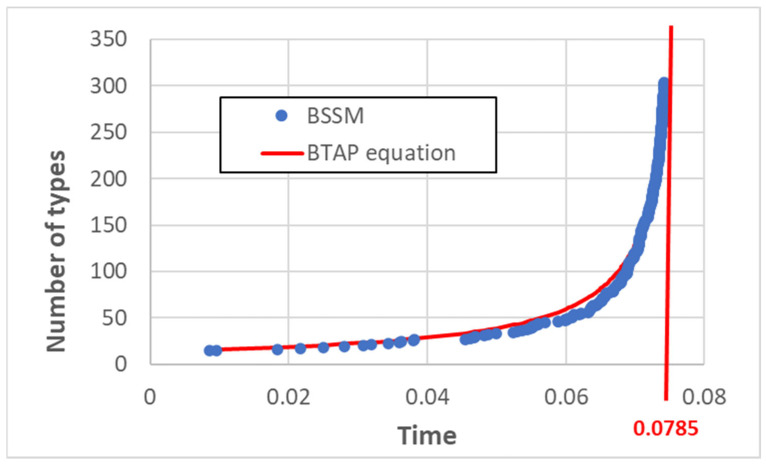
Number of existing string types in model BSSM and in the BTAP Equation (7). The values of the parameters *α*_1_ and *α*_2_, which best fit the simulation results are, respectively, 0.1 and 0.909. The corresponding divergence point of Equation (8) is also shown.

The typical behavior of the number of discovered types vs. time in a simulation of the model is shown in Figure 2. Since the algorithm is stochastic, in different realizations different species can be discovered earlier than in others. Nonetheless, the behaviors observed in these different cases are all similar, so we prefer to show an individual “typical” realization rather than average values.

Note that, if there were only cleavages, then the discovery of new types would eventually end, since cleavage cannot create new polymers longer than their parents. If the initial conditions were those described above, where all the polymers up to length three are present since the beginning, nothing would change in the cleavage-only case. If we identify the length of the longest polymer with the diameter of the model state space, then condensations can increase this diameter, while cleavages perform explorations only within its present limits. The outcome of a simulation may, therefore, depend upon the relative frequencies of cleavages and condensations, so we sometimes tested different parameter values for the occurrence probability of the two types of reactions *p_cl_* and *p_cn_*.

The behavior of a simulation of the model is also compared to that of the BTAP equation in Figure 2, showing an excellent agreement, taking into account that in simulations, no true divergence is possible, while in the BTAP model, the number of existing types of the system diverges as one approaches the time t* calculated in Equation (7).

We then tested the possible agreement of simulation results with exponential functions. As shown in Figure 2, various exponentials can fit various portions of the curve, but none are able to describe the behavior of the number of discovered types on the whole duration of the simulation. Of course, one might try fit it using linear combinations of exponentials, but this is not relevant to the argument raised in this paper. Therefore, we conclude that the growth does indeed resemble that of the BTAP.

**Figure 3:**
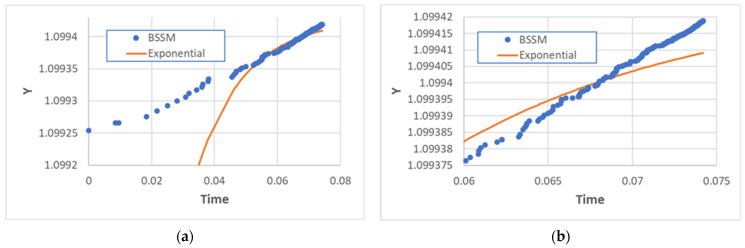
(**a**) The transformation of BSSM using Equation (10) does not show saturation, while an exponential growth of the number of types (with the same parameters of BSSM—in this case, *α*_1_ = 0.001, *α*_2_ = 0.9096, and P = 1) shows the typical behavior of a logistic function. (**b**) A magnification of the final part of (**a**), which shows that the transformation of BSSM does not show any kind of saturation in the time duration of the simulation.

We also used the criterion described in Section 2, studying the time behavior of the y variable defined in Equation (6). If the growth of *x* were TAP-like, *y*(*t*) should be an exponential function; this cannot be visually grasped from Figure 3 since, in the observed range, it is not very different from a straight line. However, it should be observed that it is indeed an increasing function, while if *x*(*t*) were exponential, then y should show saturation, and there is no sign of saturation in our simulations. Therefore, the analysis of the y function also supports the conclusion that the growth of BSSM resembles TAP, in spite of the fact that the true novelty of newborn types is checked.

## 5. Introducing Quantities

As anticipated, we are interested in exploring how sensitive the finite-time divergence may be with respect to some changes in the model presented so far. We will then consider two kinds of modifications, both implying that the probability of performing a given reaction depends upon the quantities of the corresponding reactants, which in turn depend upon how many times they have been produced in the past. The quantity of each polymer *S*, *Q_S_* is defined as the number of different reactions with *S* as a product, which have taken place so far.

Then the probability that polymer *S* undergoes cleavage is proportional to the quantity of *S*, and the probability that polymers *S* and *U* undergo condensation is proportional to the product of the corresponding quantities. In the formulae, *prob* denotes probability per unit time:

- *prob* (cleavage of *S*) = *p_cl_ Q_S_*;
- *prob* (condensation of *S* and *U*) = *p_cn_ Q_S_ Q_U_*.

Note that the rule for the probabilities of choosing a particular reaction in the BSSM model of Section 4 can be considered as a particular case of these rules, in the case where all the quantities of the existing polymers are equal to one.

In the first model variant considered in this section, which will be called BSSM2, it is assumed that reactions do not destroy reactants, just like in the BSSM model of Section 4. In this case, the quantities of reactants do not decrease if they partake as reactants in a reaction. A precise description of the BSSM2 is also given in the Appendix A.

**Figure 4:**
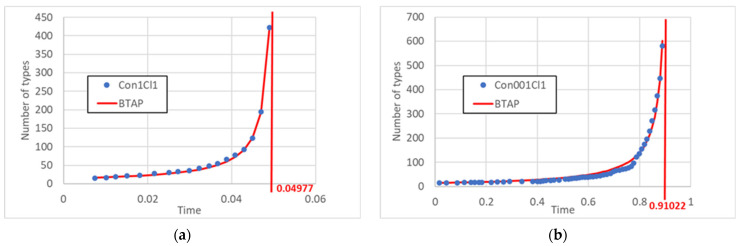
Number of existing string types in model BSSM2, interpolated by using the BTAP equation; the divergence point of Equation (7) is also shown. (**a**) Case in which the coefficients of cleavages and condensations have the same value (Con1Cl1—interpolation with the TAP equation gives *α*_1_ = 0.00108 and *α*_2_ = 1.408); (**b**) case in which the coefficients of the condensations is one hundredth of the coefficients of the cleavages (Con001Cl1—interpolation with the TAP equation gives *α*_1_ = 0.297 and *α*_2_ = 0.0585).

It would make little sense to directly compare the time behaviors of the BSSM and BSSM2 models since, in the latter case, the Gillespie algorithm computes time basing both on the reaction coefficients and the quantities of types, and the latter factor does not appear in the BSSM case. It seems more appropriate to compare these models on the basis of the number of reactions which have been performed, as done in Figure 4 below. The main result is that, despite the slowdown in the number of new types created with the same number of performed reactions in the BSSM2 case, this model still shows a TAP-like behavior, similar to the previous one. We also checked that this property is robust with respect to a change in the ratio *p_cl_/p_cn_* of the kinetic coefficients, as shown by comparing Figure 4a,b (of course, the actual rate of growth differs in the two cases).

As anticipated in Section 1, we also considered another variant of the model, called BSQM since it includes quantities, which more closely resembles chemical kinetics by assuming that reactants do indeed disappear. Therefore, the quantities of those types which are involved in a reaction diminish by following the reaction stoichiometry: if polymer *S* undergoes a cleavage reaction at time t, its quantity decreases (*Q_S_* → *Q_S_*−1). The same happens if it undergoes condensation with a different polymer. If, however, it undergoes condensation with another specimen of the same type, then *Q_S_* → *Q_S_*−2. A precise description of the BSQM is given in the Appendix A.

**Figure 5:**
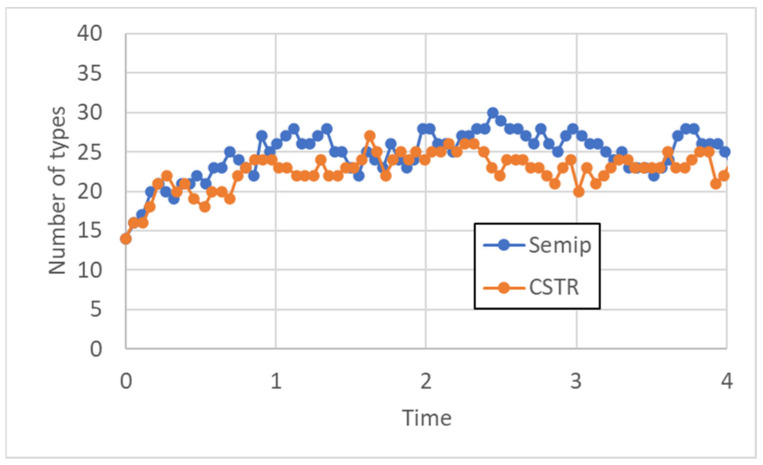
Number of chemical species present inside a CSTR and inside a container separated from the outside by a semi-permeable membrane (case “SemiP”). The CSTR flux and the environmental concentrations outside the container are set to have a final comparable number of chemical species in the two cases. The two systems start having monomers, dimers, and trimers inside; in the flow entering the CSTR there is a constant concentration of monomers “A” and “B”, the same substances, which in the “SemiP” case can pass the membrane and whose concentrations are buffered in the external environment.

Having introduced quantities, the model is no longer well-tailored to describe the dynamics of types, but it rather becomes one of population dynamics, dealing with the interactions between individual strings. In this case it is also necessary to place our system in a proper environment. An isolated system would ultimately be limited by the number of initial monomers; therefore, it is necessary to consider open systems, which can exchange matter with their environment. We have simulated two kinds of open systems, i.e., a homogeneous continuously stirred chemical flow reactor (CSTR) and a constant-volume vesicle separated from the buffered external environment by a semi-permeable membrane. We will not discuss here these two cases any further, referring the interested reader to the original papers [16,17,22] for further details. In both cases, the familiar pattern of growth of the number of polymers up to a finite saturation level is observed, as shown in Figure 5.

## 6. Conclusions

In this paper we have applied the TAP equation to the Binary String World, where the major difference, with respect to the “canonical” formula given in Equation (3), is that the expansion on the right-hand side is limited to second order terms. We have also introduced the time-continuous version of this, BTAP, showing that it can be analytically solved, and that it can diverge at finite times. We have also stressed that the TAP equation is meant to describe the dynamics of types, not of specimens (as it is done in usual models of population dynamics).

The BSW can also be simulated with a dynamical model, BSSM, which allows us to directly check whether a newborn entity, which has just been produced, is actually new, corresponding to a type never seen before. This is not possible in the canonical TAP approach, and we will call this property the “distinguishability of novelties”. Some different equations, inspired by a TAP kind of approach, have been proposed and studied [4,13], but none of them deal with the problem of the distinguishability of novelties.

The simulation of the BSSM shows a very steep growth in the number of different types. While a finite simulation cannot of course truly diverge, we showed that this growth is similar to TAP, rather than to the familiar exponential growth. Therefore, it appears, at least in the case of the BSW, that checking the distinguishability of newly generated types, and discarding those which are identical to already existing species, does not cancel the main feature of TAP, i.e., its super-exponential character. This is likely to be a major outcome of this study.

It would be interesting to explore how generic this result is, and to determine under which conditions the super-exponential growth is maintained in different models, able to distinguish true novelties. The fact that this feature is conserved also in the BSSM2 model in Section 5 suggests that it might be robust with respect to some important model changes. On the other hand, the results of BSQM prove that it cannot be found in models of population dynamics, which describe the change of the number of individuals, rather than types.

The importance of combinations for the evolution of technology has already been emphasized elsewhere [6,7,12], although the question can be raised as to which kinds of new combinations count as truly new types. It is worth mentioning that the “explosive” character of TAP shows how powerful combinations can be also in biology and suggests that this might be one of the reasons why life is based on combinations of building blocks, like amino acids or nucleotides [23]. It may be suggested that combinations of similar kinds could have been involved in the processes which led to the origin of life. The TAP equation has recently been used in [24] to show how autocatalytic sets might have arisen in prebiotic conditions. In this respect, it is intriguing to be reminded that the binary polymer model had been first introduced [Kauffman 1986] in studies about the phase of chemical evolution, which preceded the appearance of life on earth or elsewhere in the universe.

## Data Availability

Algorithm descriptions within the text are sufficient to reconstruct the work. No other data is needed.

## Appendix A

The aim of this Appendix is that of precisely describing the models which have been applied in this work to the Binary String World. While in the text the models were introduced in the order BSSM, BSSM2, and BSQM, in this Appendix we first describe BSQM, which, as mentioned in Section 5, is not dealing with types, but rather with individual entities. It is, therefore, closer to models of population dynamics or of chemical reactions, and it is amenable to be simulated with Gillespie’s algorithm, ideated to stochastically simulate sets of chemical reactions. The BSSM and BSSM2 methods will then be presented considering them as variants of the BSQM model.

**BSQM model**

Table A1 shows the data structures of the BQSM method; Table A3 shows some details of a particular procedure (“execute_reaction”, which takes care of carrying out the reaction chosen to be carried out in a given instant); Table A2 shows the general outline of BQSM method. Given an initial list of already present types, the list of possible reactions is created (in our case, all the cleavages that can be performed on the present types, and all the condensations that can be performed starting from the present types).

After that, and until the final simulation time has been reached (see Table A3):

- The probability of occurrence of each reaction is calculated, starting from the list and the number of each type (Gillespie schema);
- The single reaction that has to happen is chosen (roulette algorithm);
- The corresponding dt is calculated (Gillespie schema);
- The reaction is carried out (description below);
- The Δ*t* just calculated is added to the total time.

Carrying out a reaction involves (procedure “execute_reactionBSQM”, Table A2):

- The decrease in the number of items of the types present as reactants, following the stoichiometry of the reaction;
- The increase the number of items of the types present as products, following the stoichiometry of the reaction;
- The update to the possible reactions, eliminating those whose reactants are no longer present and, if there are new types, adding those that are now possible.

**Table A1:** Data structures of the used schema.

Data Structures	
Lreactions:	list of possible reactions.
Ltypes:	list of present types (for each type the number of items is stored).
Lreact_prob:	list of the probabilities of occurrence of each reaction.

**Table A2:** General schema. See the text for a description.

General Schema
Ltypes ← from configuration file
List_of_reactions = create_reactions(Ltypes, Lreactions)
time ← 0
while time < time_max do
Lreact_prob =compute_reaction_probability(Ltypes, Lreactions)
m = choose_reaction(Lreact_prob)
dt = compute_dt(Lreact_prob)
(Ltypes, Lreactions) = execute_ reactionBSQM(m, Ltypes, Lreactions)
time ← time + dt

**Table A3:** Procedure execute_reactionBSQM (m, Ltypes, Lreactions). Given the list of types present in the system (Ltypes), the list of reactions present (Lreactions) and the selected reaction (m), the procedure executes the reaction and returns the updated lists of types and reactions.

Execute_reactionBSQM (m, Ltypes, Lreactions): List of Calls.
decrease_reactants(m, Ltypes): the procedure decreases the number of items
of the reactants of reaction m (updates Ltypes).
adjourn_products(m, Ltypes): the procedure increases the number of items
of the products of reaction m (updates Ltypes).
create_reactions(Lreactions): the procedure updates the list of possible reactions,
starting from the list of present types (updates Lreactions).
return Ltypes, Lreactions

**BSSM algorithm**

The purpose of the BSSM algorithm is to model the creation of new types, starting from the existing types: in this process the number of instances of each type plays no role and the existing types do not disappear. As discussed in the main text, the number of types already present is influential, as it increases the number of possible reactions and as consequence the frequency of their occurrence.

To take into account these characteristics it is appropriate to use Gillespie’s scheme already presented in BSQM model, in which though (i) all reactions have the same dynamic coefficient, (ii) the types once created do not disappear, and (iii) each type is present with an item only. In this way each reaction acquires the same probability of occurrence, and the system as consequence chooses the next reaction with a uniform probability distribution. It is, therefore, sufficient to modify the procedure execute_reactionBSQM() of the previous scheme to obtain the BSSM model.

In Table A4 we present procedure execute_reactionBSSM(), which differs from execute_reactionBSQM() in that (i) the quantities of reactants are never modified (the procedure decrease_reactants() of Table A3 is absent) and (ii) the number of items of products cannot exceed the value “1” (at the time of the first production the number of items of the new type is equal to 1, and if the product is already present in the system, its value is not incremented by the adjourn_productsBSSM() procedure).

**Table A4:** Procedure execute_reactionBSSM (m, Ltypes, Lreactions). Given the list of types present in the system (Ltypes), the list of reactions present (Lreactions) and the selected reaction (m), the procedure executes the reaction and returns the updated lists of types and reactions. Note that if the product is already present in the system, its value is not incremented by the adjourn_productsBSSM() procedure.

Execute_reactionBSSM (m, Ltypes, Lreactions): List of Calls.
adjourn_productsBSSM(m, Ltypes): the procedure adds an item to the new products,
but does not change the quantities of the already existing types (updates Ltypes).
create_reactions(Lreactions): the procedure updates the list of possible reactions,
starting from the list of present types (updates Lreactions).
return Ltypes, Lreactions

**BSSM2 algorithm**

The BSSM2 model allows the number of items of each type to grow (up to a threshold TH, set equal to 10 in the simulations of this paper); reactions, on the other hand, do not consume the reactants.

In Table A5 we present the execute_reactionBSSM2() procedure, which differs from execute_reactionBSSM() in the fact that adjourn_productsBSSM2() procedure allows the number of product items to grow (following the stoichiometry of the reaction) up to a maximum of TH (once it has been reached, the number of items of that product does not increase any further).

**Table A5:** Procedure execute_reactionBSSM2(m, Ltypes, Lreactions). Given the list of types present in the system (Ltypes), the list of reactions present (Lreactions) and the selected reaction (m), the procedure executes the reaction and returns the updated lists of types and reactions. Note that the execute_reactionBSSM2() allows the number of product items to grow up to the threshold TH, while it does not cause the number of items of reactants to decrease.

Execute_reactionBSSM2 (m, Ltypes, Lreactions): List of Calls.
adjourn_productsBSSM2(m, Ltypes): the procedure allows the number of product items
to grow up to a maximum of threshold TH (updates Ltypes).
create_reactions(Lreactions): the procedure updates the list of possible reactions,
starting from the list of present types (updates Lreactions).
return Ltypes, Lreactions.
